# The vaccinologist’s “dirty little secret”: a better understanding of structure-function relationships of viral immunogens might advance rational HIV vaccine design

**DOI:** 10.1007/s00705-021-04982-7

**Published:** 2021-02-19

**Authors:** Gregor P. Greslehner

**Affiliations:** 1 ImmunoConcept, UMR5164, CNRS and University of Bordeaux, Bordeaux, France; 2 Department of Philosophy, University of Vienna, Vienna, Austria

## Abstract

I will offer a conceptual analysis of different notions of structure and function of viral immunogens and of different structure-function relationships. My focus will then be on the mechanisms by which the desired immune response is induced and why strategies based on three-dimensional molecular antigen structures and their rational design are limited in their ability to induce the desired immunogenicity. I will look at the mechanisms of action of adjuvants (thus the wordplay with Janeway’s “immunologist’s dirty little secret”). Strategies involving adjuvants and other (more successful) vaccination strategies rely on taking into account activities and functions (“what is going on”), and not just the structures involved (“who is there”), in binding in a “lock and key” fashion. Functional patterns as well as other organizational and temporal patterns, I will argue, are crucial for inducing the desired immune response and immunogenicity. The 3D structural approach by itself has its benefits – and its limits, which I want to highlight by this philosophical analysis, pointing out the importance of structure-function relationships. Different functional aspects such as antigenicity, immunogenicity, and immunity need to be kept separate and cannot be reduced to three-dimensional structures of vaccines. Taking into account different notions of structure and function and their relationships might thus advance our understanding of the immune system and rational HIV vaccine design, to which end philosophy can provide useful tools.

## Introduction

In his seminal 1989 paper, Charles A. Janeway Jr., a key figure in immunology, coined the phrase “the immunologist’s dirty little secret” [1], together with what he called the “Landsteinerian fallacy”, referring to the fact that the mere presence of a foreign antigen was not equally able and sufficient to trigger an adaptive immune response. He was convinced that, for immunogenicity, signals from host cells are required in addition to the presence of an antigen. One way in which these additional signals could be provided in a setting that did not involve the presence or activity of pathogens was by adding so-called “adjuvants”. It became common practice to add aluminum salts or other components as adjuvants, without always knowing their exact mechanism of action. Although many details have become much better understood in immunology in the decades since then [2], especially since the discovery of Toll-like receptors [3] and other pattern recognition receptors, we are still far from “approaching the asymptote” [1] in our understanding of the immune system, and many open questions remain.

In this paper, I want to look at how a better understanding of structure-function relationships can clarify some conceptual issues underlying the difficulties with developing an HIV-1 vaccine by rational design. One underlying mindset responsible for a limited perspective, according to my argument, is the neglect of some issues addressed in the previous introductory paragraph – which I will refer to as the “vaccinologist’s dirty little secret”. I will argue that the almost exclusive focus of rational vaccination design on three-dimensional molecular shapes of antigens has made it prone to difficulties similar to the ones expressed earlier by Janeway. I will then suggest a framework of structure-function relationships for understanding the scope and limits of each of the notions of structure and function as immunological targets for vaccine development and therapies of HIV/AIDS.

Vaccinations are often huge success stories, like in the case of smallpox. Other vaccination efforts against, for example, the common cold or seasonal influenza are a constant struggle and race against time. During the ongoing COVID-19 crisis, massive vaccination efforts have yielded promising results in a short time. While, at the time of writing, the widespread use of a vaccine is still at least several months away, the speed at which progress has been made in vaccine development has been impressive. On other fronts, however, vaccination attempts have been unsuccessful despite massive efforts for several decades. HIV-1 is the prime example of a virus – and its accompanying disease – for which it has not yet been possible to develop a vaccine. There are a number of reasons, which will be addressed throughout this special issue (see also references [4, 5]). In my paper, I want to focus on one particular aspect that constitutes a central puzzle for (much of) biology in general and poses a major challenge to so-called “rational vaccination design” against HIV-1 in particular: structure-function relationships.

Both terms – ‘structure’ and ‘function’ – have multiple meanings we need to distinguish first before we can draw meaningful relationships between these notions. After offering a conceptual analysis of the terms and their respective meanings, we will address the kinds of relationships that can – and cannot – be claimed to exist between them. There are a number of general conceptual and theoretical ramifications beyond rational vaccination design, but this is a field in which misunderstandings about structure-function relationships might have been underlying some major obstacles in attempting to design a vaccine rationally. These reasons will be the main focus of the paper.

## ‘Structure’ and ‘function’ are polysemic terms

Most biology textbooks feature the slogan “structure determines function”. Its exact meaning and scope, however, are less than clear. Before addressing the relationships between structure and function, let’s take a closer look at the terms themselves. Most prominently, when thinking about proteins and other molecules, and especially when it comes to components of the immune system, ‘structure’ usually refers to the three-dimensional shape of molecules (str_3D_). This str_3D_, in turn, is said to be determined by its amino acid sequence – another kind of structure (str_seq_). The sequence of amino acids, again, depends on the corresponding str_seq_ of nucleotides; first DNA, which is then transcribed into RNA and consequently translated into protein. From a simple reductionist view, but originally phrased in terms of “information”, this belongs to the so-called “central dogma of molecular biology” [6].

$${\text{DNA }} \to {\text{ RNA }} \to {\text{ protein}}$$

In a next step, we have the widespread belief that a protein’s structure will determine its function. Thus, according to the reductionist ideal, knowing the genetic code would allow us to predict all of the protein structures and functions encoded in a genome. Unfortunately, this linear chain of determination relations, ${\text{str}}_{{{\text{seq}}}} \to {\text{ str}}_{{{\text{3D}}}} \to {\text{ function}}$, does not hold. Besides the intricacies of how nucleotides get translated and transcribed into protein, the protein folding problem still poses a major challenge, and intrinsically unstructured proteins [7] and multifunctional “moonlighting” proteins [8] further defy this linear chain of determination.

While these high expectations have been disappointed for many reasons, one fundamental issue remaining is the actual meaning of the word ‘function’ and how much of a genome should be considered to be functional or “junk” [9, 10]. In this context, it is very useful to distinguish between different notions of function. Philosophers have argued for decades about different concepts and meanings of the word ‘function’ [11, 12]. The two biggest classes of alternative accounts originate back to Robert Cummins' “causal role” account [13] and Larry Wright’s “selected effects” notion of function [14]. Disputes between these camps continue to this day, but in the briefest way I can put it, the causal role account aims at explaining *how* something can do what it does, whereas the selected effects account strives to illuminate *why* an organism’s trait, capacity or feature can do what it does. I believe that these are two very different explanatory goals that justify having different notions of function. However, when they are confused for each other, they give rise to serious misunderstandings. A conceptual analysis with philosophical tools can be helpful for avoiding these misunderstandings.

A very useful distinction of four different notions has been suggested by Arno G. Wouters [15]: On the one hand, we have function as biochemical activity (fct_act_), and on the other hand, we have the biological role (fct_role_) as a functional contribution to a complex capacity at a higher level of organization. These cannot be linked to a single entity or activity, but rather to an entire mechanism network of entities and activities, which together give rise to the functional aspect of the biological role in question. In short, fct_role_ cannot be explained at the level of str_3D_ and fct_act_; it can only be explained with reference to another kind of organizational network structure (str_org_). In addition to these two causal role notions of function, there are two more evolutionary considerations of function (fct_evol_) to be distinguished: biological advantage and selected effect. Since we are not concerned with evolutionary considerations in this paper, I will refer to both of these aspects as “fct_evol_”. Especially when it comes to the relationships of structures and functions, evolutionary questions will be beyond the physiological aspects of the other notions of structure and function. Instead, they address why some of them show a different persistence by natural selection. On the physiological scale, however, higher levels of str_org_ can easily be imagined: from intra- and intercellular signaling networks and physiological mechanisms to social networks of populations, whose dynamics play a major role in the control of diseases in networks with community structure.

## Relationships between different notions of structure and function

With this distinction of various notions of structure and function in mind, it is time to address their relationships. As briefly discussed earlier, the reductionist ideal of a linear chain of determination from sequence to structure to function does not work. Protein folding predictions have been quite successful due to the fact that the folding space appears to be quite modular with a number of repeated folding motifs [16]. Usually, a high degree of sequence similarity is a good indication of a similar str_3D_. The successful prediction of the biochemical activity of a protein usually depends on knowledge of the activity of a protein with a very similar str_3D_. Even though not a perfect proxy, it is a usually a good indicator for a conserved protein domain with similar biochemical properties and thus activities in a different protein. Its biological role, however, cannot be predicted solely by its str_3D_ and comparison to similar str_3D_. Think, for example, of all the hundreds of different kinases. While they all share the same basic biochemical activity, i.e., catalyzing phosphorylation, their biological roles can be completely different [17], depending on the interaction network str_org_ they are part of – and their dynamics.

A similar distinction can be made between antigenicity and immunogenicity, the confusion of which contributes to the conceptual problems of developing a structure-based HIV-1 vaccine [4, 18]. Attempts to develop vaccines, or to understand and intervene in biological systems in general, based solely on predictions from str_3D_ is inherently limited. Once the different structure-function relationships have been spelled out in detail, many problems and misunderstandings can be clarified. The hope to predict a molecule’s ability to trigger an immune response and provide long-term immunity solely based on its str_3D_ and binding capacities (fct_act_) is in vain – although it is a central assumption in structure-based reverse vaccinology attempts.

Table 1 provides an overview of the different notions and their relations. All of them are exception-ridden and face some non-trivial problems, defying the simple reductionist ideal of being determined from the “lower-level” notions. Pitting these levels or notions against each other as more fundamental would be a fruitless enterprise. In the same way, all of the different aspects and notions of structure and function are important for explaining different phenomena; it is important to have a molecular and systems perspective [19]. This is a general lesson for biology, and it is particularly important for interventions aiming at eliciting appropriate immune responses and long-term immunity in individuals and populations.

**Table 1:** Different notions of structure and function, between which a meaningful relationship can be established. ‘∆str/fct’ refers to evolutionary changes in structure or function.

Notion of structure or function	Relates to	Kind of relation
str_seq_	str_3D_	Genetic code, protein folding
str_3D_	fct_act_	Correlation, modular build of protein domains
str_org_	fct_role_	Network dynamics, systems biology
∆str/fct	fct_evol_	Natural selection, fitness advantage

## Antigenicity ≠ immunogenicity ≠ immunity

Why do the intricate details of these structure-function relationships matter for vaccination design? One major obstacle in the attempts to develop such a “bottom-up”, reductionist, structure-based vaccine [20, 21] rests on the simplistic, unwarranted assumption that str_3D_ can be linked straightforwardly to fct_act_ and fct_role_. Failing to acknowledge the difference between the biochemical activity (fct_act_) and the biological role (fct_role_), such as epitope binding and immunogenicity, results in misunderstandings and neglect of immunological theory [22]. As Marc H. V. Van Regenmortel put it:

The reductionist mindset made immunologists accept that the biological activities of Abs [antibodies] could be explained by their 3D structures and that the immunogenic potential of a viral epitope could be deduced from its antigenic properties. Biological immunogenicity was thereby reduced to chemical antigenicity, which is a variation of the claim that biology can be reduced to chemistry [...]. Such a claim fails to recognize that the protection achieved by vaccination is a biological phenomenon that has meaning only in the context of an entire organism since organs, tissues, or molecules cannot be vaccinated. Protection always results from a complex network of dynamic interactions between pathogen, host, and immune system and it cannot be satisfactorily understood when innumerable, individual molecular interactions are analyzed separately. [23]

With the proposed distinction of different notions of structure, function, and their structure-function relationships, it is easy to see why the underlying fallacy is a confusion of different categories of functions. The same is true for the level of structures involved, as he also rightly points out that molecules cannot be vaccinated. The target of vaccination and the level at which immunogenicity can take place is not str_seq_ or str_3D_, it is str_org_ [20, 24].

The problems may lie even deeper, with a focus on things rather than the underlying processes. Only the activities and functions result in antigenicity, immunogenicity, and protective immunity. These cannot be reduced to antigenicity, let alone be an intrinsic property of or be explained solely by any particular kind of structure. For that very reason, perhaps a *process ontology* perspective [25] is thus better suited for thinking about these issues in immunology. A similar case is being made to consider viruses as processes rather than things [26]. For the current question, however, we will stick with structure-function relationships and the general lesson that the functions (processes) of antigenicity, immunogenicity, and immunity we are interested in cannot be reduced to any structures. Structures should not be considered more fundamental than functions. Neither should these different functions be confused with each other.

In addition, the notion of immunogenicity itself is something worth reconsidering conceptually [27]. Rather than basing immunological distinctions on notions of self and non-self, the spatio-temporal patterns of entities and their activities, a discontinuity of which would be the trigger for an immune response [28, 29], might be a better criterion for immunogenicity than the mere presence of a molecular str_3D_ that we would classify as “foreign” or “dangerous”. And finally, immunogenicity, i.e., the successful induction of an immune response, is still not the same as the protective immunity that vaccines are ultimately aiming for. These issues need to be addressed in the complex context of both individual immune systems and populations – str_org_ at different levels of organization and their functions.

With the distinction between fct_act_ and fct_roles_, we can clearly see why str_3D_ can be linked (imperfectly, with correlations) to fct_act_, such as antigenicity – even though there are also some non-trivial problems. However, a fct_role_ such as immunogenicity or protective immunity, which we are ultimately interested in when developing a vaccine, cannot be meaningfully linked to just fct_act_ or str_3D_. If at all, str_org_ would need to be considered to understand the operations of immune systems at the level of their complex interaction networks. With systems immunology and systems vaccinology still in their infancy, we are far from such an extent of knowledge and understanding to venture with such an approach. It is doubtful whether the required completeness of knowledge about complex immune system interactions will ever be of an adequate quality to understand, let alone design, vaccines based on the str_org_ of these networks and their components and activities.

Although it is a different kind of reductionism than basing everything solely on str_3D_, there is not much reason to be too optimistic about such attempts succeeding in the near future in developing HIV vaccines. It is not out of the question that major steps will be made in that direction, but it will be rather by “accident”, i.e., empirically by trial and error rather than based on knowing the primitive structural and functional components of these immune systems. Being aware of the distinctions and different relationships will be a helpful conceptual tool in avoiding such misconceptions and embarking on research paths that are most likely inherently doomed to fail. With this negative prospect of what has not worked in the past and the underlying conceptual problem of structure-function relationships, which alternatives might work? And how might a better understanding of structure-function relationships help in these endeavours?

## Can empirical approaches be more successful?

Due to the evasive nature of HIV-1 structures and functions as a target for vaccination design, especially if designed rationally “bottom-up” with an emphasis on str_3D_, other alternatives might provide more expedient routes to developing a vaccine – or even ones with more promise of success: envisage treatment and cures. On the other hand, the most efficient way to stop the HIV/AIDS pandemic would still be a vaccine. While other approaches to preventing and treating HIV infection are showing fascinating and encouraging results, finding a vaccine remains a central challenge. In order to be successful, empirical approaches rather than “rational” design based on str_3D_ might be more expedient. Reductionist, simplistic assumptions about structure-function relationships might be misleading and at the root of many failed approaches in the past [20, 30]. Despite the success of molecular, and especially structural, biology, there are a number of theoretical limits, which do not allow for the reductionist ideal to “explain *all* biology in terms of physics and chemistry” [31].

Returning to the “dirty little secret” of immunologists or vaccinologists, adjuvants such as aluminum hydroxide (or alum, for short) [32, 33] and their functions in addition to the str_3D_ of antigens have to be taken into account – together with the str_org_ of complex interaction networks on different levels of organization. Once again, the processes of fct_act_ and fct_role_ are responsible for the outcome, not just the structures that are involved.

An interesting observation^1^ is the recent development of nanofiber vaccines that work without adjuvants [34]. While the underlying mechanisms are not yet completely understood, it is suspected that the mode of antigen delivery plays a crucial role in inducing immunity. The same is probably true for other adjuvant-free vaccines, such as some seasonal flu vaccines. Once again, this can be interpreted in a conceptual framework where functions, not just structures, are an important factor for being recognized by the immune system and triggering an immune response [35].

With a better understanding of the working mechanisms of vaccine adjuvants [36, 37] – what they are, or are not, needed for – and of structure-function relationships, vaccination design could be able to combine empirical and rational approaches. In addition to adjuvants, there may be a number of other (unknown) factors that are also important for immunogenicity. Once we acknowledge the fct_act_ and fct_role_ of adjuvants, they are “no longer a dirty little secret, but essential key players in vaccines of the future” [38]. Instead of neglecting immunological theories [22] and using outdated concepts of antibody specificity [23], future vaccination attempts should focus on notions of structure and function other than just str_3D_. Systems biology approaches to vaccination are a promising first step in this direction [39–41].

Taken together, the story of adjuvants and the mechanisms by which they function underscores that, for the immune system, “what is going on” might be more important than “who is there”, i.e., the functions rather than the structures could be the relevant difference makers for whether or not an immune response is triggered and whether or not a vaccine is successful in establishing immunity. In other words: processes instead of things [25]. For all of these alternative approaches and for keeping the relevant processes apart, an improved understanding of structure-function relationships should be helpful – at the very least, to avoid falling for certain conceptual misunderstandings that take a simplistic, reductionist view on structure and function.

## Conclusion and outlook

Binding specificity, a central topic long neglected by philosophers of science, is now finally receiving more attention [42], especially with respect to drug design and HIV treatment [43]. In the past, immunology and vaccination design have put too much emphasis on str_3D_ and steric complementarity, together with the concepts of causal specificity. Structure-based vaccination design faces some fundamental conceptual limits, neglecting the complexity [44] and importance of structure-function relationships. When approaching these and other central challenges in contemporary life sciences, it is useful to remember Orgel’s second rule: “evolution is cleverer than you are”. Instead of only trying to rationally design and reverse-engineer vaccines, taking advantage of nature’s complex structures and functions to study the relationships between them and their modes of operation must not be neglected.

Thus, a better understanding of structure-function relationships might advance rational HIV vaccine design, open up other pathways of prevention and therapy, and help avoiding theoretical misconceptions that have posed a barrier in other areas of biology and medicine. Providing the required conceptual analysis is a way in which philosophy can contribute to such scientific problems [45] – in particular, when there are calls for a change of “paradigm” [46, 47]. While many “secrets” remain to be uncovered in vaccinology – and immunology in general – addressing them conceptually allows us to tackle them with novel experiments and empirical approaches; this way, they can inspire new lines of research rather than remaining “dirty little secrets”.

## Footnotes

1 Thanks to reviewer 1 for bringing this to my attention.
